# Predicting MiRNA-Disease Association by Latent Feature Extraction with Positive Samples

**DOI:** 10.3390/genes10020080

**Published:** 2019-01-24

**Authors:** Kai Che, Maozu Guo, Chunyu Wang, Xiaoyan Liu, Xi Chen

**Affiliations:** 1 School of Computer Science and Technology, Harbin Institute of Technology, Harbin 150001, China; chekai@hit.edu.cn (K.C.); chunyu@hit.edu.cn (C.W.); liuxiaoyan@hit.edu.cn (X.L.); xi.chen@stu.hit.edu.cn (X.C.); 2 School of Electrical and Information Engineering, Beijing University of Civil Engineering and Architecture, Beijing 100044, China; 3 Beijing Key Laboratory of Intelligent Processing for Building Big Data, Beijing 100044, China

**Keywords:** microRNAs, disease, association prediction, latent feature extraction

## Abstract

In discovering disease etiology and pathogenesis, the associations between MicroRNAs (miRNAs) and diseases play a critical role. Given known miRNA-disease associations (MDAs), how to uncover potential MDAs is an important problem. To solve this problem, most of the existing methods regard known MDAs as positive samples and unknown ones as negative samples, and then predict possible MDAs by iteratively revising the negative samples. However, simply viewing unknown MDAs as negative samples introduces erroneous information, which may result in poor predication performance. To avoid such defects, we present a novel method using only positive samples to predict MDAs by latent features extraction (LFEMDA). We design a new approach to construct the miRNAs similarity matrix. LFEMDA integrates the disease similarity matrix, the known MDAs and the miRNAs similarity matrix to identify potential MDAs. By introducing miRNAs and diseases knowledge as the auxiliary variables, the method can converge to give the optimal solution in each iteration. We conduct experiments on high-association diseases and new diseases datasets, in which our method shows better performance than that of other methods. We also carry out a case study on breast neoplasms to further demonstrate the capacity of our method in uncovering potential MDAs.

## 1. Introduction

MicroRNAs (miRNAs), a class of small endogenous non-coding RNAs, regulate gene expression at a post-transcriptional level through mRNA degradation or translational inhibition [1,2,3]. There is growing evidence that miRNAs are essential in biological process including immunoreaction, transcription, proliferation, differentiation, signal transduction, embryonic development and so on [4,5,6,7,8,9]. miRNA mutation, biosynthesis and dysfunction with the miRNAs of its targets can lead to various diseases [10,11,12,13]. Therefore, it is very important to identify the association between miRNAs and diseases. Early studies determined the relationship between miRNAs and specific diseases via biological experiments. However, biological experiment methods have long cycles and high costs. Therefore, computational biological methods for analyzing and predicting the association between miRNAs and diseases have been receiving great attention. 

Currently, the association prediction of miRNAs and diseases has two main categories: one based on network topology, and the other based on machine learning methods. Network topology methods are based on the observation that diseases regulated by functional similar miRNAs are similar and vice versa [14,15]. A series of research works is predicated on this hypothesis [16,17,18,19,20,21,22,23,24]. In 2010, Jiang et al. [16] first proposed a method to predict the association between miRNAs and diseases by constructing functional-related miRNAs networks and human disease phenotype-miRNA networks, and then ranking the human miRNAs according to their disease-related scores. This is a reasonable extension of using network-based methods to predict protein coding genes related to diseases. To improve their previous work, Jiang et al. [17] believed that more data sources should be introduced to increase credibility, and they proposed a new method based on genomic data fusion. They use a naive Bayesian model to fuse multiple data sources, and constructed a model to predict the functional relevance between genes. The association between diseases and genes, and between miRNAs and target genes, are represented by two vectors respectively. For a given disease, its similarity scores with all miRNAs are calculated and ranked in descending order. The miRNA with the highest score is the target associated with the disease. Chen et al. [18] provided another, more time-efficient idea which applies the random walk algorithm (RWRMDA) to the miRNA-miRNA functional similarity network. Starting from a given seed node, in order to mine the potential association in the network, the size of the known associations is used as the transfer probability to simulate the process in which the miRNA-disease relationship binding with the current node is propagated to its neighboring nodes. In 2013, Chen et al. [19] expanded the application scope and strategy comparison of different similarities on the basis of the previous article, and they proposed a similarity-based approach that consists of three strategies: microRNA-based similarity inference (MBSI), phenotype-based similarity inference (PBSI) and network-consistency-based inference (NetCBI). Shi et al. [20] proposed a new method based on restart random walk with the restart (RWR) algorithm in 2013, which maps disease genes and miRNA target genes to protein-protein interaction (PPI) networks and sets different seeds to apply the RWR algorithm. This method introduces the protein data source as the intermediary information, which improves the accuracy and credibility of the model. Since then, different researchers have experimented with multiple data sources and similarity strategies to predict miRNA and disease relationships. Later, Xuan et al. [21] proposed a method called “HDMP” to predict disease-related miRNAs based on weighted, most similar k-nearest neighbors. Xu et al. [22] predicted cancer-associated miRNAs by comparing the phenotypic association of multiple diseases between miRNAs and mRNAs expression profiles. In 2013, Mork et al. [23] proposed a protein-mediated prediction method that predicts the association between miRNAs and diseases through the association between miRNAs and proteins and the association between proteins and diseases. In 2016, Sun et al. [24] proposed a method based on the known topological similarity of miRNA disease to discover more potential disease-related miRNAs. In this method, they used bipartite projection to complete the correlation prediction. Up to now, the methods based on network topology tend to use the known association to mine the potential association. Since it lacks miRNAs and diseases with known relationship information, the results incline to be random.

The second category of methods is found on machine learning. In 2012, Xu et al. [25] first used machine learning methods to predict the relationship between miRNAs and diseases. This approach aims to identify positive associations from large-scale negative associations. The core of this method is to extract features from the miRNA-disease network and train an SVM classifier. In 2013, Jiang et al. [26] constructed the different feature sets including miRNA and a disease phenotype information set, and it achieved a similar result compared with the method of Xu. In 2014, Chen et al. [27] considered a semi-supervised global approach (regularized least squares for miRNA-disease association, RLSMDA) to predict the miRNAs-diseases associations without negative samples. This method is also applicable to diseases with unknown related miRNAs. Machine learning-based approaches can attain similar or better results than network-based topology approaches, and some approaches even handle diseases with unknown miRNAs, such as RLSMDA. Chen et al. [28] proposed an approach to combine miRNAs/diseases’ statistical feature profile and graph theoretical feature profile for MDA prediction. The idea is to project them onto the same subspace, during which Laplacian regularization is used to preserve the data’s local structures and important miRNA/disease features are selected with L-1 norm constraint. This model is promising in the way that feature selection helps dramatically reduce the dimensionality, and thus enables easy extension to higher dimensional datasets. However, these methods are mainly limited to the feature representation of miRNAs and diseases. You et al. [29] proposed a novel path-based method for MDA prediction (PBMDA). In addition to conventional two similarity and one association, a Gaussian interaction profile kernel is further introduced to measure the similarity between miRNAs and diseases. With all four statistics, a heterogeneous graph consisting of three interlinked sub-graphs is constructed, and then the depth-first search algorithm is applied on it to infer potential MDAs. The algorithm based on matrix factorization solves the problem of feature representation by using high-dimensional space vectors. It constructs the representation of miRNAs and diseases in high-dimensional space at the same time, and then obtains their association. The probability of the final miRNAs-diseases association is solved by the least square method. Such an idea is derived from the widely-adopted method of matrix factorization in recommendation systems. It has been proved that the method is very effective to solve association prediction problems in recent years. Shen [30] first proposed a matrix factorization based method (CMFMDA) to predict miRNAs-diseases associations in 2017. This approach achieved better performance than Chen [27]. However, due to the impact of its loss function, the least squares method cannot be used in the process of iteration. To a large extent, the result depends on the initial value. In many cases, it is difficult to guarantee the stability of the algorithm because it may not converge. Besides, the approaches based on matrix factorization regard the unlabeled association as the negative samples. Thus, they extract the wrong information, which leads to result deviation. A recent study by Chen et al. [31] presents the first decision tree learning-based model for MDA prediction (EGBMMDA). Obeying the routine of integrating the miRNA functional similarity, the disease semantic similarity, and known MDAs, the model uses statistical measures, graph theoretical measures, and each miRNA-disease pair’s matrix factorization result to form an informative feature vector. With calculated feature vectors and known associated pairs, a regression tree is trained under the gradient boosting framework, which is further used for predicting potential MDAs.

This paper proposes a novel approach called miRNA-disease association prediction using latent feature extraction with positive samples (LFEMDA). First, we design a new miRNAs functional similarity construction method to solve the problem that miRNAs functional similarity is used to predict miRNAs-Disease associations, while sometimes the former is dependent on the latter, which is not desirable in common inference models. Second, LFEMDA introduced miRNAs and disease knowledge as the auxiliary variables so that the optimal solution can be obtained in each iteration of the optimization process. Third, LFEMDA uses only positive samples for feature extraction, and it could reduce the deviation. Finally, LFEMDA achieves great results on both the high-association diseases data and the new diseases data.

## 2. Materials and Methods 

### 2.1. Disease Semantic Similarity Network

The disease semantic similarity is calculated by the method of Wang [32], which depends on their common semantic annotations and shared disease symptoms. Every disease can be represented by a directed acyclic graph (DAG), and a disease *D* is denoted as DAG(*D*) = (*TD*, *ED*), where *TD* is a set that includes all the ancestor nodes of *D* and *D* itself, and *ED* is a set of direct linking edges of *D*. The node *t* (t∈TD) is defined as follows:

(1) $$C_{D}(t)=\left\{ \begin{array}{ll} 1, & if\;t=D\\ \max\{\Delta \times C_{d}(t^{\prime })|t^{\prime }\in children\;of\;t\}, & if\;t\neq D, \end{array}\right.$$

where Δ is an semantic contribution factor. We set it to 0.5, as suggested in literature [24]. The semantic value of *D, DV(D)* is defined as:

(2) $$DV(D)=\sum_{t\in TD}C_{D}(t)$$

The more terms in DAG are shared between two diseases, the more similar they are. So the semantic similarity between disease *d1* and disease *d2* is defined as follows:

(3) $$\mathrm{SD}(d1,d2)=\frac{\sum_{t\in T(d1)\cap T(d2)}\left(C_{d1}(t)+C_{d2}(t)\right)}{\sum_{t\in T(d1)}C_{d1}(t)+\sum_{t\in T(d2)}C_{d2}(t)}$$

### 2.2. miRNAs-Disease Association Network

We get the miRNA-disease association information from a HMDD database [33]. The data contains 10,381 experimentally-confirmed associations among 378 diseases and 571 miRNAs. Matrix *R* represents the associations between miRNAs and diseases. For example, if miRNA *m_i_* and disease *d_j_* are related, the value *R(m_i_, d_j_)* is 1, and 0 otherwise.

### 2.3. miRNAs Functional Similarity Network

Based on the assumption that miRNAs with similarity functions are involved in similar diseases, Wang et al. [32] gave a method to get functional similarity between two miRNAs by calculating the similarity between two groups of diseases that are associated with them respectively. Cui et al. developed a tool called MISIM based on Wang’s method [32] to measure the pairwise functional similarity of the given miRNAs. MISIM can be downloaded from http://www.cuilab.cn/files/images/cuilab/misim.zip. Usually, the two disease groups are obtained from miRNAs-Disease associations. However, it leads to a problem that the miRNAs functional similarity used to predict miRNAs-Disease Associations can be actually implied by the prediction target themselves. That is to say, the miRNAs functional similarity is inferred from miRNAs-disease association and disease semantic similarity, but such inferred results may be, in reverse, incorporated in the process of predicting miRNAs-disease association.

To deal with this issue, we designed a new algorithm to obtain the functional similarity of miRNAs from their sequence data. The sequence of miRNA determines its uniqueness and function, so our method can reserve the biological characteristics to the greatest extent.

We defined the functional similarity of two miRNAs as *SM (m_1_, m_2_)*.

(4) $$SM(m_{1},m_{2})=1-\frac{Levenshtein^{\prime }(m_{1},m_{2})}{len(m_{1})+len(m_{2})}$$

$Levenshtein^{\prime }(m_{1},m_{2})$ refers to the editing distance of two miRNA sequences. So, we have,

(5) $$0\leq Levenshtein^{\prime }(m_{1},m_{2})\leq len(m_{1})+len(m_{2})$$

The miRNAs functional similarity matrix can be obtained by calculating the functional similarity between miRNAs. Suppose, for example, we have two miRNA sequences. One is hsa-mir-21(CAACACCAGUCGAUGGGCUGU), the other is hsa-mir-155(CUCCUACAUAUUAGC AUUAACA). Their *Levenshtein* distance is 19, and the functional similar score is 1 − 19/(21 + 22) = 0.5581.

### 2.4. Data Fusion

The final miRNAs similarity matrix (*MS*) and disease similarity matrix (*DS*) are obtained by integrating the miRNAs functional similarity network, the diseases semantic similarity network, and the experimentally-confirmed miRNA-disease association network (*R*). After fusing the above three datasets, there are 446 miRNAs, 322 diseases and 5,512 confirmed miRNA-disease associations in reserve. 

### 2.5. Loss Function

In this paper, the idea of matrix decomposition is used to solve the problem of miRNA-disease association prediction. Let *MS* represent the miRNAs functional similarity network, *DS* represent the diseases semantic similarity network, and *RS* represent the miRNA-disease association network.

Firstly, for each miRNA and disease, we give the initial projection vector in a fixed *k* dimension space, and use their inner product to represent the association between them, which can be denoted as follows:

(6) $$R^{\prime }=M^{T}D$$

where *M* is a m×k matrix, and *m* is the number of miRNAs. Similarly, *D* is a k×d matrix, and *d* is the number of diseases. The goal is to minimize the distance between $R^{\prime }$ and the real relationship *R* by solving the appropriate *M* and *D*, which can be expressed as:

(7) $$\min{\left\|R^{\prime }-R\right\|}_{F}^{2}$$

Only the positive samples are credible, so the Formula (5) can be described as:

(8) $$\min{\sum_{R_{i,j}=1}\left(R^{\prime }{}_{i,j}-R_{i,j}\right)}^{2}$$

In addition, the constrained *M* and *D* are hoped to match the priori *MS* and *DS* in the model, so the loss function can be written as:

(9) $$\min\lambda_{1}{\left\|MM^{T}-MS\right\|}_{F}^{2}+\lambda_{2}{\left\|DD^{T}-DS\right\|}_{F}^{2}$$

Considering the terms $M_{i}\times M^{T}$ and $D_{j}\times D^{T}$, the quadratic form may exist in the loss function. This prevents us from getting a simplified equation about the interested variables during the optimization, which will make it impossible to get the optimal solution in the iteration process. So, matrix *X* and *Y* are introduced as the auxiliary variables to help the optimization. The Formula (9) is transformed as:

(10) $$\min\lambda_{1}{\left\|MX^{T}-MS\right\|}_{F}^{2}+\lambda_{2}{\left\|DY^{T}-DS\right\|}_{F}^{2}+\mu_{1}{\left\|M-X\right\|}_{F}^{2}+\mu_{2}{\left\|D-Y\right\|}_{F}^{2}$$

Empirically, two-norm constraints are added on *M* and *D* to prevent the model falling into overfitting. The final loss function is as follows:

(11) $$\begin{array}{ll} L= & \sum_{R_{i,j}=1}\left({\left(M_{i}D_{j}{}^{T}-R_{i,j}\right)}^{2}\right)\;+\lambda_{0}\left({\left\|M\right\|}_{F}^{2}+{\left\|D\right\|}_{F}^{2}\right)+\lambda_{1}{\left\|MX^{T}-MS\right\|}_{F}^{2}\\ & +\lambda_{2}{\left\|DY^{T}-DS\right\|}_{F}^{2}+\mu_{1}{\left\|M-X\right\|}_{F}^{2}+\mu_{2}{\left\|D-Y\right\|}_{F}^{2} \end{array}$$

### 2.6. Optimization

For Formula (11), there are four variables in the loss function, so there is no method to solve the optimal *M* and *D* directly. Thus, we use an iterative least squares approach to get its optimal solution. At the same time, since only positive samples participate in the optimal process, it is hard to optimize the function by matrix calculation. To settle this problem, it will solve the hidden variable of each miRNA and disease. The specific steps are as follows:

Firstly, using current *D, X, Y* to update *M*. Take the derivative of *M_i_*:

(12) $$\begin{array}{l} \frac{\partial L}{\partial M_{i}}=2\cdot \sum_{R_{i,j}=1}\left(\left(M_{i}D_{j}{}^{T}-R_{i,j}\right)\cdot D_{j}\right)+\lambda_{0}\cdot M_{i}+\lambda_{1}\cdot \left(M_{i}X^{T}-MS_{i}\right)\cdot X+\mu_{1}\left(M_{i}-X_{i}\right)\\ =2\cdot \sum_{R_{i,j}=1}\left(M_{i}D_{j}{}^{T}D_{j}-R_{i,j}D_{j}\right)+\lambda_{0}\cdot M_{i}+\lambda_{1}\cdot M_{i}X^{T}X-\lambda_{1}\cdot MS_{i}X+\mu_{1}M_{i}-\mu_{1}X_{i} \end{array}$$

Let $\frac{\partial L}{\partial M_{i}}=0$, and then we can get:

(13) $$\begin{array}{c} M_{i}=\left(\sum_{R_{i,j}=1}R_{i,j}D_{j}+\lambda_{1}\cdot MS_{i}\cdot X+\mu_{1}\cdot X_{i}\right){\left(\sum_{R_{i,j}=1}D_{j}{}^{T}D_{j}+\left(\lambda_{0}+\mu_{1}\right)\cdot I_{k}+\lambda_{1}\cdot X^{T}X\right)}^{-1}\\ =\left(\sum_{R_{i,j}=1}D_{j}+\lambda_{1}\cdot MS_{i}\cdot X+\mu_{1}\cdot X_{i}\right){\left(\sum_{R_{i,j}=1}D_{j}{}^{T}D_{j}+\left(\lambda_{0}+\mu_{1}\right)\cdot I_{k}+\lambda_{1}\cdot X^{T}X\right)}^{-1} \end{array}$$

Similarly, fixing other parameters and solving *D*, *X*, and *Y* respectively:

(14) $$D_{j}=\left(\sum_{R_{i,j}=1}M_{i}+\lambda_{2}\cdot DS_{i}\cdot Y+\mu_{2}\cdot Y_{i}\right){\left(\sum_{R_{i,j}=1}M_{i}{}^{T}M_{i}+\left(\lambda_{0}+\mu_{2}\right)\cdot I_{k}+\lambda_{2}\cdot Y^{T}Y\right)}^{-1}$$

(15) $$X=\left(\lambda_{1}\cdot MS^{T}\cdot M+\mu_{1}\cdot M\right){\left(\lambda_{1}\cdot M^{T}M+\mu_{1}I_{k}\right)}^{-1}$$

(16) $$Y=\left(\lambda_{2}\cdot DS^{T}\cdot D+\mu_{2}\cdot D\right){\left(\lambda_{2}\cdot D^{T}D+\mu_{2}I_{k}\right)}^{-1}$$

Thus, the optimal solution of *M, D, X* and *Y* is obtained. This process will be iterated until it converges.

### 2.7. Prediction

We use the inner product of calculated *M* and *D* to obtain a new correlation matrix $R^{\prime }=M^{T}D$, and $R^{\prime }(i,j)$ is the predicted association of the *i*th miRNA and the *j*th disease. In fact, the value of $R^{\prime }(i,j)$ makes sense only when compared with other values in the matrix $R^{\prime }$. The larger the value, the higher possibility that associations exist. But it is not equal with the probability of existential association. The specific steps of the LFEMDA algorithm are shown in Algorithm 1. The code and data of LFEMDA is freely available at https://raw.githubusercontent.com/kavinche/fantastic-telegram/master/data_and_code_of_LFEMDA.rar.

## 3. Results and Discussion

### 3.1. Performance Evaluation

We adopted Leave-One-Out Cross-Validation (LOOCV) to evaluate the performance of our approach and other miRNA-disease association prediction methods. For each known miRNA-disease association, it is left out in turn as the test data. All the other known associations are treated as training data. The unknown associations are regarded as candidates. After the association prediction, each pair of disease and miRNA will get a score; the larger the score, the greater the probability of association.

**Algorithm 1:** LFEMDA, predicting miRNA-disease association by latent feature extraction with positive samples**Input:***MS*: m*m miRNAs functional similarity matrix    *DS*: d*d disease semantic similarity matrix    *R*: the experimentally confirmed miRNAs-disease association matrix **Paramter:** k: hidden space dimension    $\lambda_{0}$: second normal form regularization coefficient    $\lambda_{1}$: the distance coefficient between expression matrix inner product of miRNAs on the hidden space and *MS*
   $\lambda_{2}$: the distance coefficient between expression matrix inner product of diseases on the hidden space and *DS*
   $\mu_{1}$: the distance coefficient between expression matrix of miRNAs on the hidden space and auxiliary matrix *X*
   $\mu_{2}$: the distance coefficient between expression matrix of diseases on the hidden space and auxiliary matrix *Y***Output:**$R^{\prime }$: the predicted miRNAs-disease association matrix Initialize the vector matrices M, D, and the auxiliary vectors X, Y of miRNAs and diseases Δ←∞, loss←∞ while Δ>ε:    update M, given current D, X and Y, using Formula (11)    update D, given current M, X and Y, using Formula (12)    calculate current X based on the new M    calculate current Y based on the new D    calculate loss_new using Formula (9)    Δ←loss_new−loss
   loss←loss_new
End while $R^{\prime }=M^{T}D$


With a predefined threshold, if the score of associated miRNA is larger than the threshold, it is considered as a correctly identified positive sample. Otherwise, it is regarded as a true identified negative sample. Then, the TPR, FPR and Receiver Operating Characteristics (ROC) can be calculated. Finally, the area under the ROC curve (AUC) is selected to measure the performance of the prediction method.

**Figure 1:**
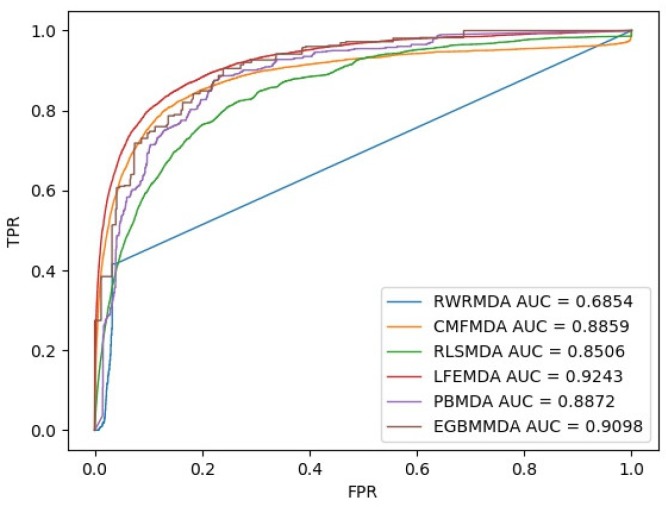
Performance comparison between LFEMDA and the other three models (RWRMDA, CMFMDA, RLSMDA, PBMDA and EGBMMDA) in terms of ROC curve and AUC based on LOOCV, respectively.

To illustrate the performance of LFEMDA, we compared it with the existing state-of-the-art methods: RWRMDA, CMFMDA, RLSMDA, PBMDA and EGBMMDA. Figure 1 is the comparison result. The hyperparameters in experiment are set as follows: $\lambda_{0}$= 6.0, $\lambda_{1}$= 0.1, $\lambda_{2}$= 0.1, $\mu_{1}$= 3.0, $\mu_{2}$= 3.0. As is demonstrated in the result, LFEMDA has the highest prediction performance among the compared methods.

### 3.2. Case Study

In Data Fusion section, there are 21 diseases which have more than 60 known associated miRNAs. Here, we can regard them as high association diseases. LFEMDA was compared with RWRMDA, CMFMDA, RLSMDA, PBMDA and EGBMMDA by LOOCV. The AUC results are showed in Table 1. The average AUCs of LFEMDA, RWRMDA, CMFMDA, RLSMDA, PBMMDA and EGBMMDA are 85.22%, 60.40%, 80.08% 63.75%, 76.33% and 82.38% respectively. LFEMDA shows the best performance on 14 high association diseases compared with other methods, and it gets better results on other 7 high association diseases. 

At the same time, to evaluate LFEMDA performance on new diseases, we compared it with RWRMDA, CMFMDA, RLSMDA, PBMDA and EGBMMDA on 20 diseases having only one known experimentally related miRNA. The experimental result is showed in Table 2. LFEMDA obtained the satisfactory results. Overall, it can be seen that LFEMDA shows excellent results not only on high association diseases, but also on new disease. EGBMMDA and PBMDA get the best results in two situations. The experimental results of CMFMDA are not unsatisfactory in six new diseases, i.e., Moyamoya Disease, Hypoxia-Ischemia Brain, Liver Diseases Alcoholic, Amyotrophic Lateral Sclerosis, Pemphigus Benign Familial and Neuroma Acoustic. For RLSMDA, it performs well on new diseases but poorly on high association diseases.

To further prove the performance of LFEMDA, a case study on breast neoplasms was carried out to demonstrate the prediction ability. Here, we used LFEMDA to identify potential miRNAs related to breast neoplasms. In addition, the prediction results were validated by three miRNA-disease association databases: HDMM, dbDEMC2 [34] and miR2Disease [35]. The top 50 breast-neoplasms-related miRNAs are listed in Table 3. The HDMM and the dbDEMC2 databases have confirmed that all the 50 predicted miRNAs are associated with the disease. The database miR2Disease have identified 47 predicated miRNAs.

### 3.3. Control Experiment with Different miRNA Functional Similarity

**Figure 2:**
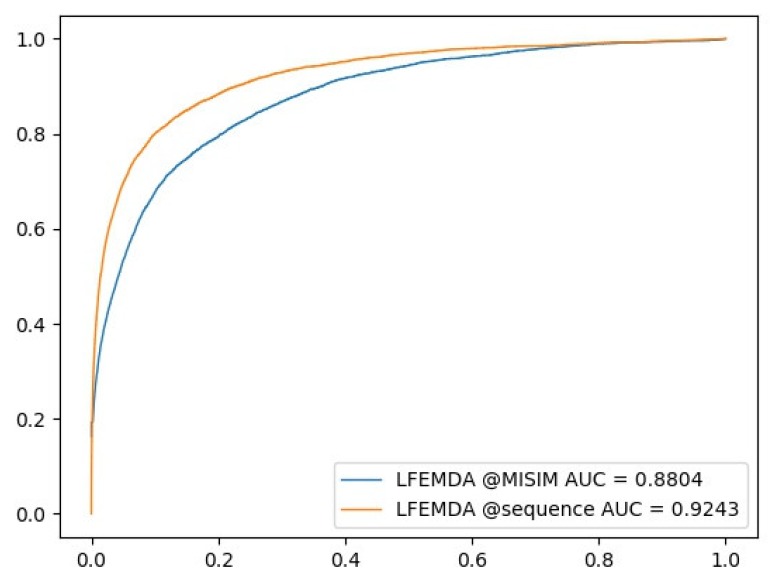
LFEMDA with different miRNA functional similarity.

In Section 2.3, we describe the reason that we designed a new miRNAs functional similarity computing method in detail. The method to calculate the miRNAs functional similarity scores is usually dependent on the miRNAs-Disease associations, and then the scores and disease semantic similarity are used to predict the associations. To avoid the scores hidden from the association information, we put forward a method to calculate the miRNAs functional similarity by miRNAs sequences. We compare the approaches with different miRNAs functional similarities. One similarity is achieved by our method, the other from MISIM. The result is displayed in Figure 2. The AUC of LFEMDA with our similarity is 92.43%, and that with similarity from MISIM is 88.04%. This illustrates the effectiveness of our method.

## 4. Conclusions

In this paper, we present a miRNA-disease association prediction method using latent feature extraction with positive samples (LFEMDA). Leave-One-Out Cross-Validation (LOOCV) is used to evaluate the performance of LFEMDA and other methods. The experiment results reveal that our method is better than others, not only on the high-association diseases data, but also on the new diseases data. The case study on breast neoplasms further demonstrates the extraordinary ability of our method to predict the potential associations. In addition, the control experiment proves that our calculation of miRNA functional similarity is effective. Regarding these contributions, we believe that LFEMDA is helpful in providing the potential candidates for subsequent research in the etiology and pathogenesis of complex diseases.

## Figures and Tables

**Table 1:** The AUC results of high-association diseases under different algorithms.

Name	Associations	LFEMDA	RLSMDA	CMFMDA	RWRMDA	PBMDA	EGBMMDA
Carcinoma, Hepatocellular	209	**0.777074623**	0.562371401	0.726182159	0.718837107	0.726162356	0.751014475
Breast Neoplasms	188	0.825615501	0.575863451	0.779712619	0.785302831	0.744000495	**0.83387968**
Stomach Neoplasms	166	0.781357444	0.596336681	0.730033905	0.415019763	0.742491394	**0.783024957**
Colorectal Neoplasms	143	**0.815816439**	0.577177998	0.771634237	0.726338286	0.764776478	0.806896074
Melanoma	133	**0.837661607**	0.627245105	0.775708519	0.809683177	0.758005237	0.820990175
Lung Neoplasms	125	**0.907236486**	0.593028386	0.860728224	0.636684303	0.79835514	0.880834891
Heart Failure	118	0.807582407	0.570803132	0.718811527	0.5	0.747054568	**0.820457834**
Neoplasms	116	**0.919919468**	0.641564425	0.873431701	0.691700345	0.82531348	0.82318139
Ovarian Neoplasms	113	**0.891470921**	0.626313211	0.843754711	0.839827262	0.768077812	0.798872832
Prostatic Neoplasms	111	0.856723686	0.628673046	0.810308089	0.795159194	0.741024607	**0.914524556**
Carcinoma, Renal Cell	100	0.843903179	0.605379769	0.786606069	0.5	0.735028902	**0.904940339**
Glioblastoma	99	**0.816009776**	0.587858911	0.785139551	0.485119944	0.791925014	0.799697261
Pancreatic Neoplasms	98	0.907911933	0.626861054	0.866743742	0.733275142	0.799173118	**0.924964814**
Carcinoma, Non-Small-Cell Lung	92	**0.869584575**	0.596822501	0.83466099	0.843493976	0.768944977	0.860077377
Urinary Bladder Neoplasms	89	**0.855515442**	0.624616265	0.796487867	0.607213439	0.735907846	0.795801467
Colonic Neoplasms	82	**0.873166319**	0.630208504	0.801865631	0.5	0.764004288	0.855367194
Carcinoma, Squamous Cell	78	**0.878461503**	0.579512281	0.832002776	0.5	0.761775362	0.815635452
Glioma	73	**0.91383381**	0.630672274	0.816208746	0.5	0.786771457	0.787616145
Esophageal Neoplasms	68	0.780247066	0.558603262	0.761111226	0.5	0.704365079	**0.790071584**
Leukemia, Myeloid, Acute	67	**0.860503335**	0.604397968	0.827720767	0.5	0.818965857	0.816735049
Head and Neck Neoplasms	63	**0.876705038**	0.63878347	0.817123175	0.800857458	0.746404741	0.715606114

**Table 2:** The AUC results for new diseases under different algorithms.

Name	Associations	LFEMDA	RLSMDA	CMFMDA	RWRMDA	PBMDA	EGBMMDA
Distal Myopathies	1	**1**	0.993258427	0.988764045	0.5	0.943820225	0.957303371
Moyamoya Disease	1	0.993258427	**0.995505618**	0.08988764	0.5	**0.995505618**	0.982022472
Hypoxia-Ischemia, Brain	1	**0.991011236**	0.988764045	0.096629213	0.5	0.901123596	0.982022472
Hypopharyngeal Neoplasms	1	0.991011236	**1**	0.838202247	0.5	**1**	0.982022472
Hepatitis C, Chronic	1	**1**	**1**	**1**	0.5	0.991011236	0.959550562
Lipid Metabolism Disorders	1	**0.993258427**	0.979775281	0.914606742	0.5	0.991011236	0.959550562
Liver Diseases, Alcoholic	1	**0.930337079**	0.739325843	0.051685393	0.5	0.824719101	0.817977528
Adenoma	1	**1**	**1**	0.930337079	0.5	**1**	0.982022472
Amyotrophic Lateral Sclerosis	1	0.95505618	0.948314607	0.11011236	0.5	0.943820225	**0.957303371**
Keratoconus	1	**1**	0.993258427	0.986516854	0.5	0.912359551	0.959550562
Aortic Aneurysm, Abdominal	1	**1**	**1**	0.964044944	0.5	**1**	0.982022472
Carcinoma, Embryonal	1	**0.865168539**	0.838202247	0.856179775	0.5	0.694382022	0.817977528
Oligodendroglioma	1	**0.907865169**	0.817977528	0.905617978	0.5	0.84494382	0.817977528
Carcinoma, Ductal, Breast	1	**1**	**1**	0.914606742	0.5	**1**	0.982022472
Fanconi Anemia	1	**0.824719101**	0.730337079	0.820224719	0.5	0.471910112	0.438202247
Colitis	1	**1**	0.997752809	0.898876404	0.5	0.997752809	0.982022472
Eye Abnormalities	1	**0.993258427**	0.82247191	0.779775281	0.5	0.912359551	0.959550562
Pemphigus, Benign Familial	1	0.991011236	**0.993258427**	0.103370787	0.5	0.970786517	0.982022472
Neuroma, Acoustic	1	0.995505618	**1**	0.173033708	0.5	**1**	0.982022472
Creutzfeldt-Jakob Syndrome	1	**1**	0.997752809	0.44494382	0.5	0.995505618	0.982022472

**Table 3:** The top 50 breast neoplasms-related miRNAs.

Rank	Name	Evidence	Rank	Name	Evidence
1	hsa-mir-21	HDMM, dbDEMC2, miR2Disease	26	hsa-mir-148a	HDMM, dbDEMC2, miR2Disease
2	hsa-mir-126	HDMM, dbDEMC2, miR2Disease	27	hsa-let-7c	HDMM, dbDEMC2, miR2Disease
3	hsa-mir-17	HDMM, dbDEMC2, miR2Disease	28	hsa-mir-34b	HDMM, dbDEMC2, miR2Disease
4	hsa-mir-34a	HDMM, dbDEMC2, miR2Disease	29	hsa-mir-182	HDMM, dbDEMC2, miR2Disease
5	hsa-mir-155	HDMM, dbDEMC2, miR2Disease	30	hsa-mir-125b-2	HDMM, dbDEMC2, miR2Disease
6	hsa-mir-20a	HDMM, dbDEMC2, miR2Disease	31	hsa-mir-30a	HDMM, dbDEMC2, miR2Disease
7	hsa-mir-146a	HDMM, dbDEMC2, miR2Disease	32	hsa-mir-19a	HDMM, dbDEMC2, miR2Disease
8	hsa-mir-34c	HDMM, dbDEMC2, miR2Disease	33	hsa-let-7d	HDMM, dbDEMC2, miR2Disease
9	hsa-mir-29a	HDMM, dbDEMC2, miR2Disease	34	hsa-mir-92a-1	HDMM, dbDEMC2
10	hsa-mir-145	HDMM, dbDEMC2, miR2Disease	35	hsa-mir-200a	HDMM, dbDEMC2, miR2Disease
11	hsa-mir-218-1	HDMM, dbDEMC2	36	hsa-mir-222	HDMM, dbDEMC2, miR2Disease
12	hsa-mir-16-2	HDMM, dbDEMC2	37	hsa-mir-143	HDMM, dbDEMC2, miR2Disease
13	hsa-mir-221	HDMM, dbDEMC2, miR2Disease	38	hsa-mir-210	HDMM, dbDEMC2, miR2Disease
14	hsa-let-7b	HDMM, dbDEMC2, miR2Disease	39	hsa-mir-31	HDMM, dbDEMC2, miR2Disease
15	hsa-mir-16-1	HDMM, dbDEMC2, miR2Disease	40	hsa-mir-375	HDMM, dbDEMC2, miR2Disease
16	hsa-mir-125b-1	HDMM, dbDEMC2, miR2Disease	41	hsa-let-7f-2	HDMM, dbDEMC2, miR2Disease
17	hsa-mir-146b	HDMM, dbDEMC2, miR2Disease	42	hsa-mir-29b-1	HDMM, dbDEMC2, miR2Disease
18	hsa-let-7a-2	HDMM, dbDEMC2, miR2Disease	43	hsa-let-7f-1	HDMM, dbDEMC2, miR2Disease
19	hsa-mir-10b	HDMM, dbDEMC2, miR2Disease	44	hsa-let-7e	HDMM, dbDEMC2, miR2Disease
20	hsa-mir-200b	HDMM, dbDEMC2, miR2Disease	45	hsa-let-7g	HDMM, dbDEMC2, miR2Disease
21	hsa-mir-200c	HDMM, dbDEMC2, miR2Disease	46	hsa-mir-27a	HDMM, dbDEMC2, miR2Disease
22	hsa-mir-218-2	HDMM, dbDEMC2, miR2Disease	47	hsa-mir-181a-2	HDMM, dbDEMC2, miR2Disease
23	hsa-mir-22	HDMM, dbDEMC2, miR2Disease	48	hsa-mir-30c-2	HDMM, dbDEMC2, miR2Disease
24	hsa-mir-18a	HDMM, dbDEMC2, miR2Disease	49	hsa-mir-25	HDMM, dbDEMC2, miR2Disease
25	hsa-mir-133b	HDMM, dbDEMC2, miR2Disease	50	hsa-mir-486	HDMM, dbDEMC2, miR2Disease
